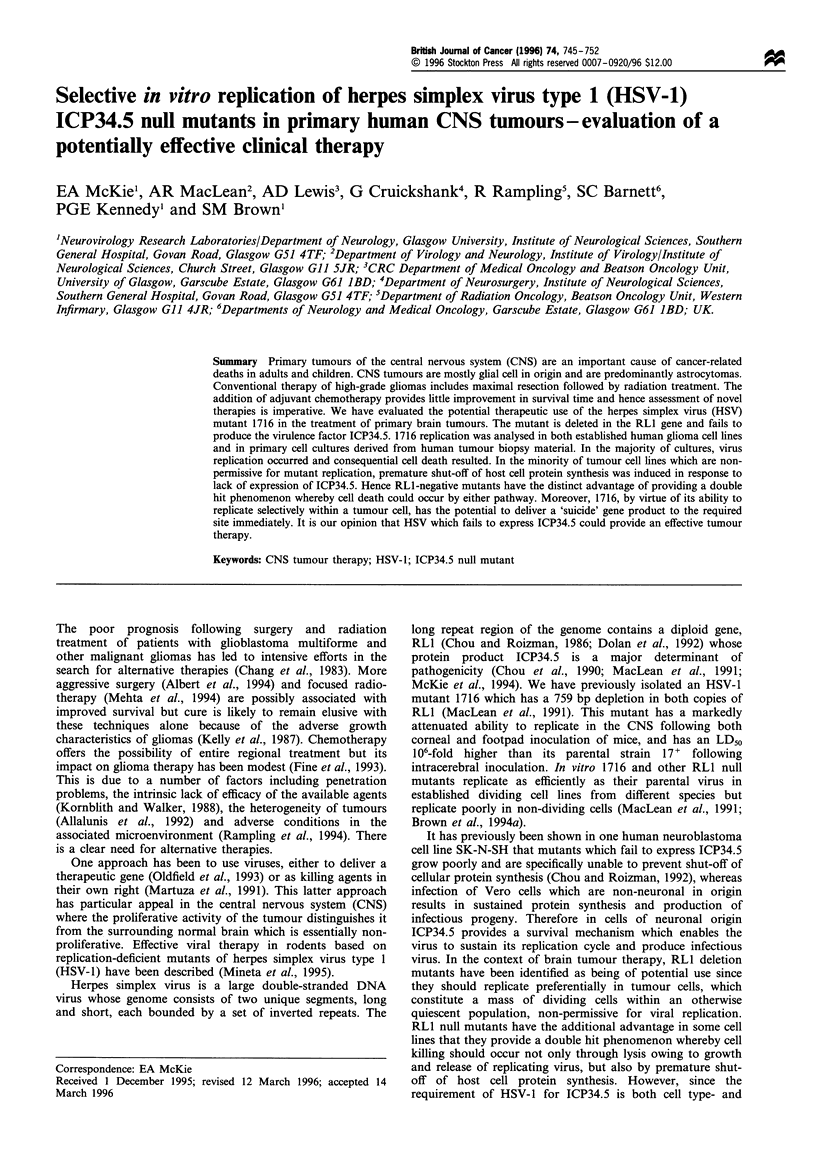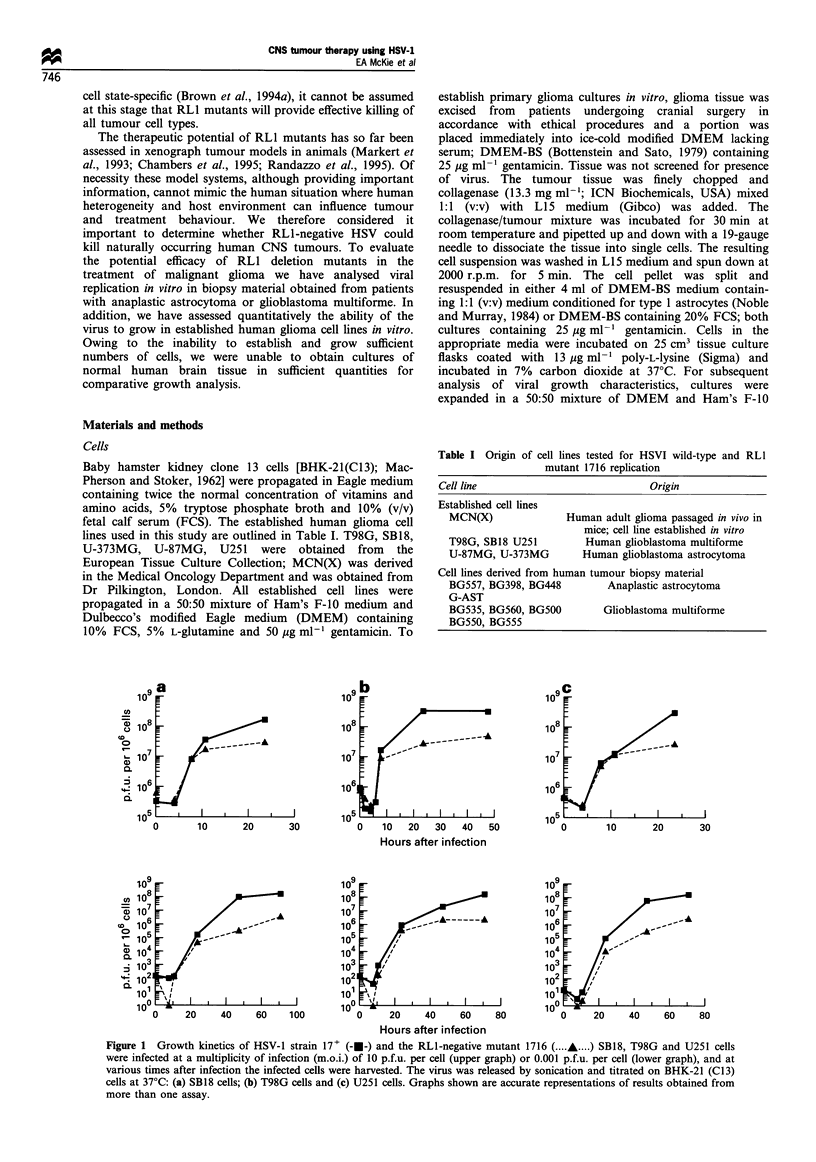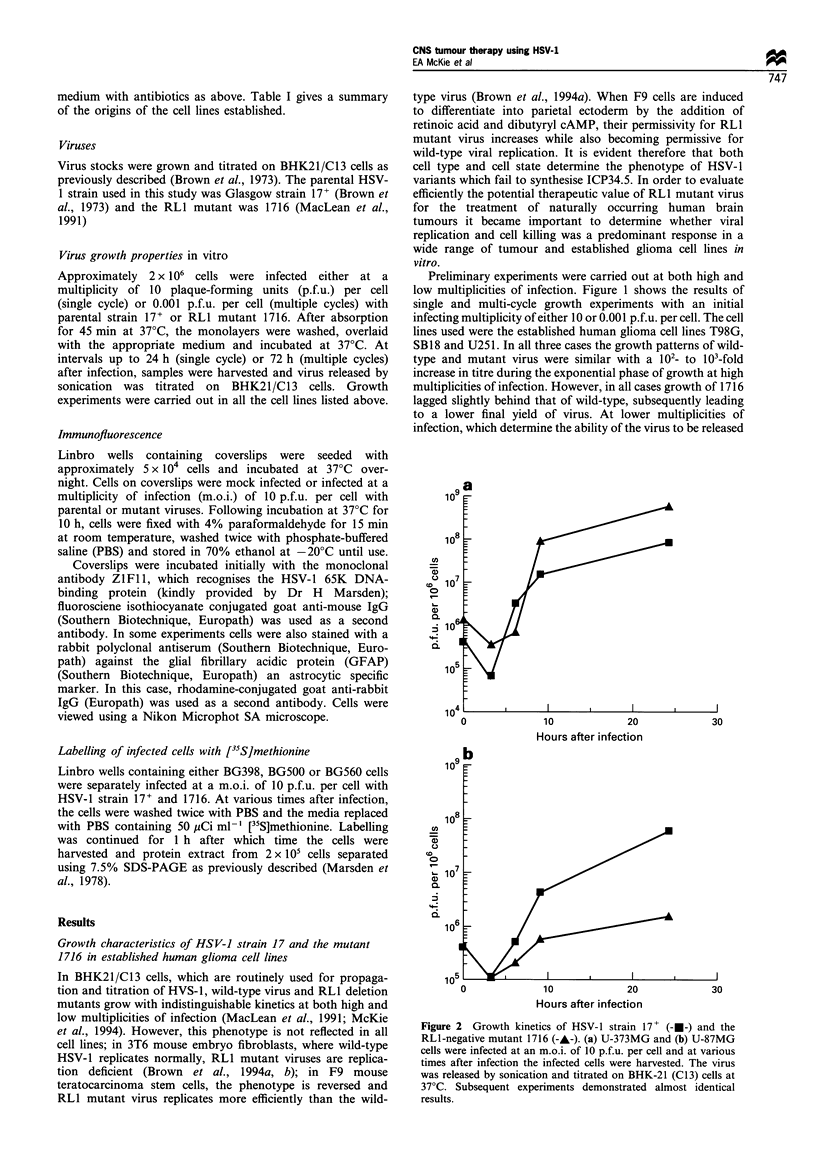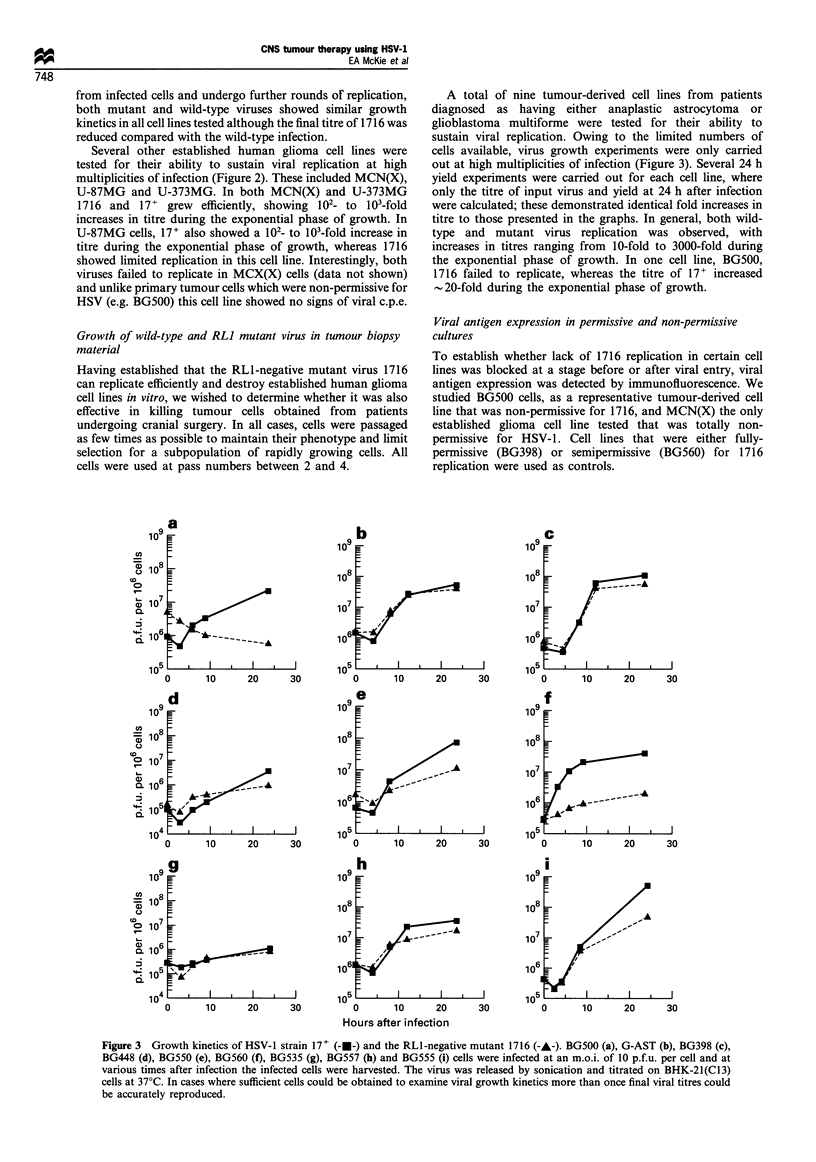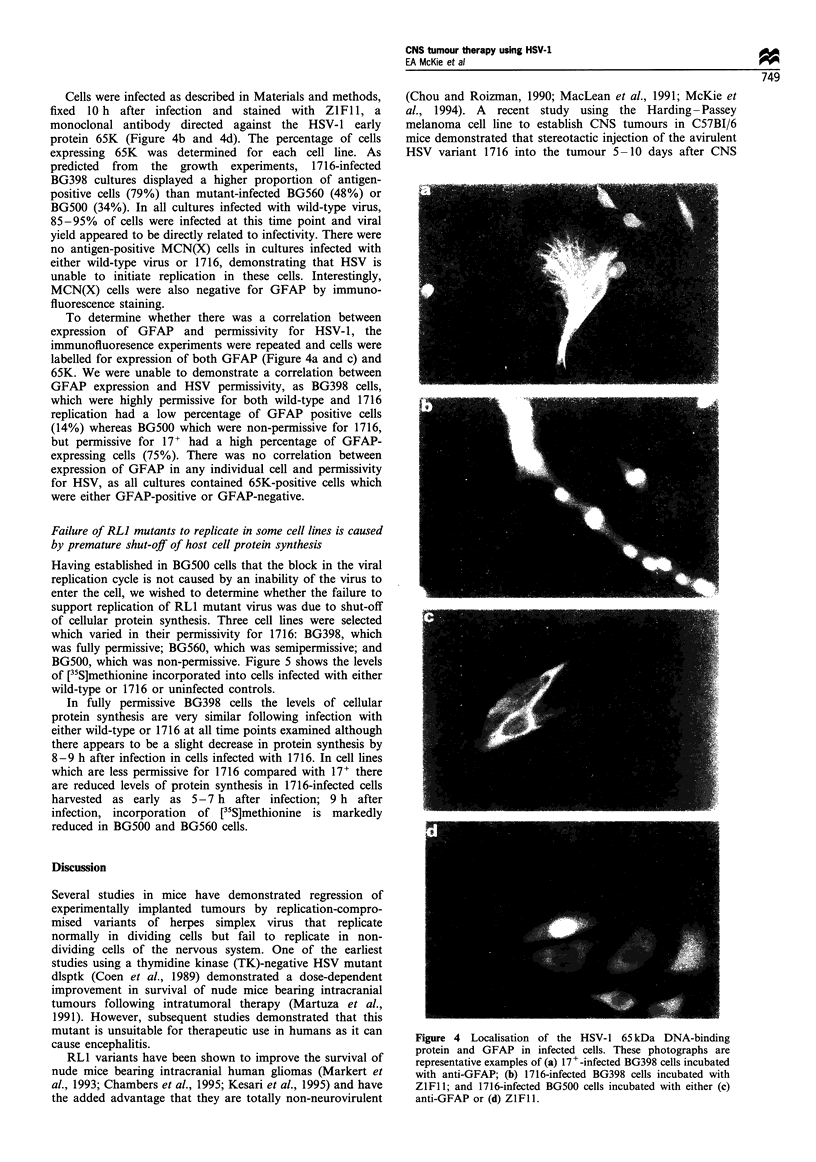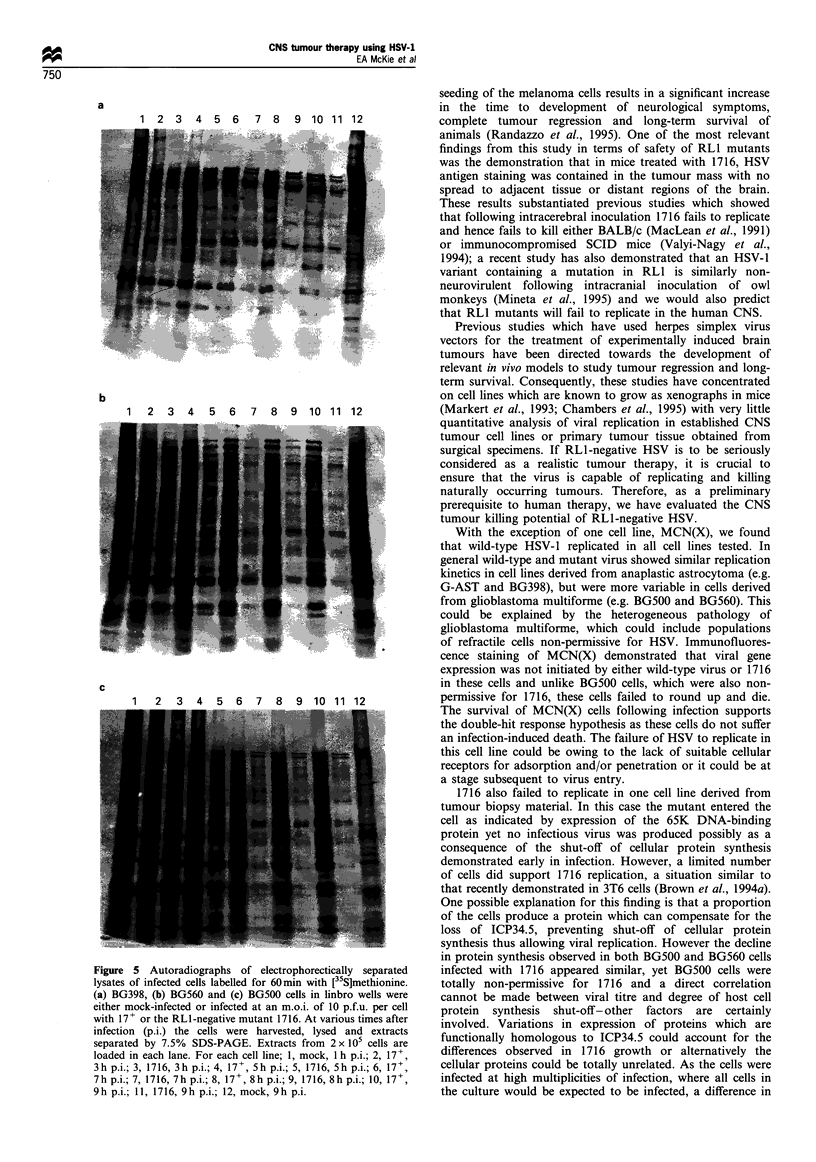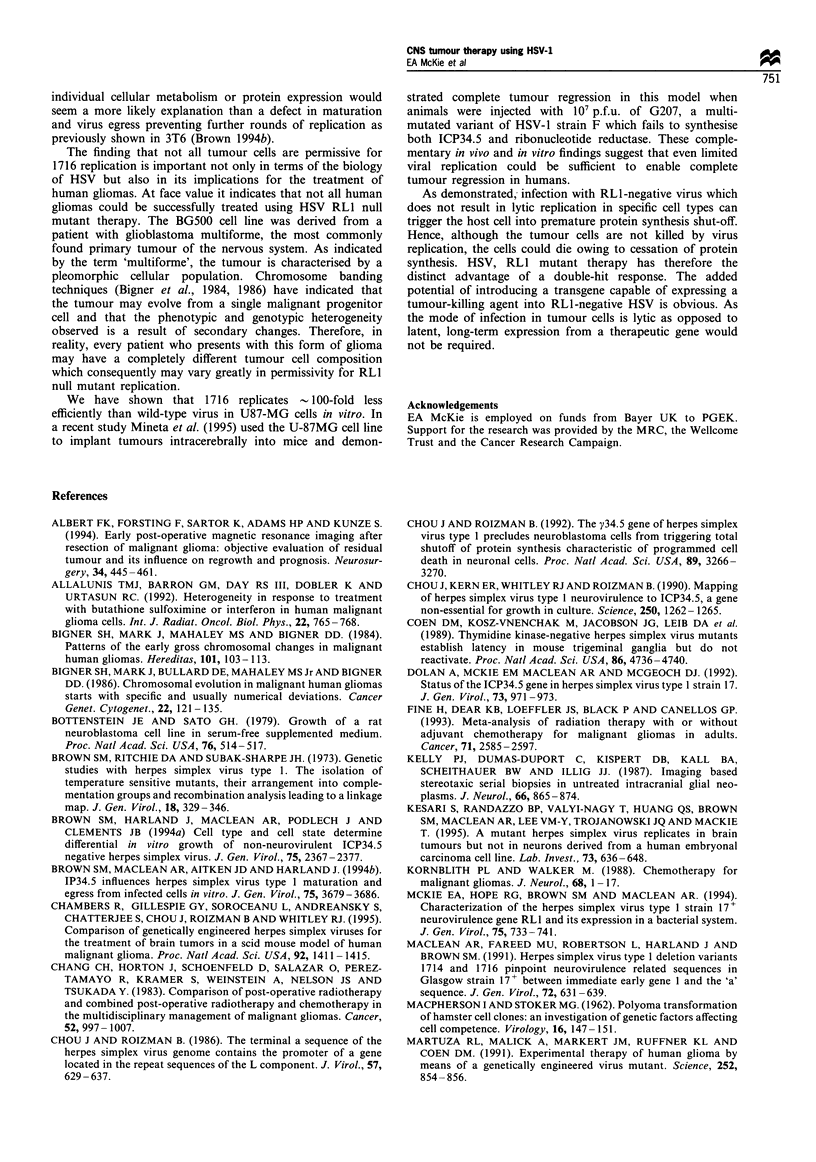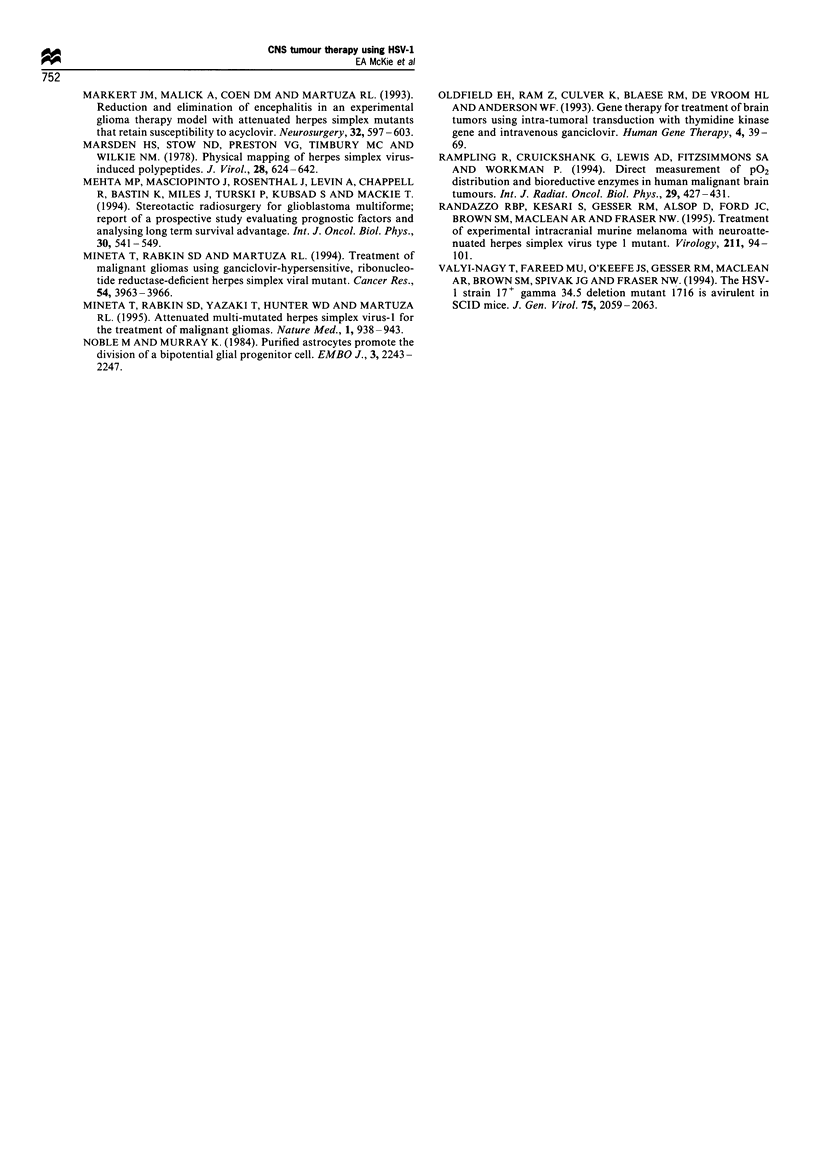# Selective in vitro replication of herpes simplex virus type 1 (HSV-1) ICP34.5 null mutants in primary human CNS tumours--evaluation of a potentially effective clinical therapy.

**DOI:** 10.1038/bjc.1996.431

**Published:** 1996-09

**Authors:** E. A. McKie, A. R. MacLean, A. D. Lewis, G. Cruickshank, R. Rampling, S. C. Barnett, P. G. Kennedy, S. M. Brown

**Affiliations:** Neurovirology Research Laboratories/Department of Neurology, Glasgow University, UK.

## Abstract

**Images:**


British Journal of Cancer (1996) 74, 745-752
© 1996 Stockton Press All rights reserved 0007-0920/96 $12.00

### Selective *in vitro* replication of herpes simplex virus type 1 (HSV-1) ICP34.5 null mutants in primary human CNS tumours – evaluation of a potentially effective clinical therapy

EA McKie[1], AR MacLean[2], AD Lewis[3], G Cruickshank[4], R Rampling[5], SC Barnett[6], PGE Kennedy[1] and SM Brown[1]

*[1]Neurovirology Research Laboratories/Department of Neurology, Glasgow University, Institute of Neurological Sciences, Southern General Hospital, Govan Road, Glasgow G51 4TF; [2]Department of Virology and Neurology, Institute of Virology/Institute of Neurological Sciences, Church Street, Glasgow G11 5JR; [3]CRC Department of Medical Oncology and Beatson Oncology Unit, University of Glasgow, Garscube Estate, Glasgow G61 1BD; [4]Department of Neurosurgery, Institute of Neurological Sciences, Southern General Hospital, Govan Road, Glasgow G51 4TF; [5]Department of Radiation Oncology, Beatson Oncology Unit, Western Infirmary, Glasgow G11 4JR; [6]Departments of Neurology and Medical Oncology, Garscube Estate, Glasgow G61 1BD; UK.*

**Summary** Primary tumours of the central nervous system (CNS) are an important cause of cancer-related deaths in adults and children. CNS tumours are mostly glial cell in origin and are predominantly astrocytomas. Conventional therapy of high-grade gliomas includes maximal resection followed by radiation treatment. The addition of adjuvant chemotherapy provides little improvement in survival time and hence assessment of novel therapies is imperative. We have evaluated the potential therapeutic use of the herpes simplex virus (HSV) mutant 1716 in the treatment of primary brain tumours. The mutant is deleted in the RL1 gene and fails to produce the virulence factor ICP34.5. 1716 replication was analysed in both established human glioma cell lines and in primary cell cultures derived from human tumour biopsy material. In the majority of cultures, virus replication occurred and consequential cell death resulted. In the minority of tumour cell lines which are non-permissive for mutant replication, premature shut-off of host cell protein synthesis was induced in response to lack of expression of ICP34.5. Hence RL1-negative mutants have the distinct advantage of providing a double hit phenomenon whereby cell death could occur by either pathway. Moreover, 1716, by virtue of its ability to replicate selectively within a tumour cell, has the potential to deliver a 'suicide' gene product to the required site immediately. It is our opinion that HSV which fails to express ICP34.5 could provide an effective tumour therapy.

**Keywords:** CNS tumour therapy; HSV-1; ICP34.5 null mutant

The poor prognosis following surgery and radiation treatment of patients with glioblastoma multiforme and other malignant gliomas has led to intensive efforts in the search for alternative therapies (Chang *et al.*, 1983). More aggressive surgery (Albert *et al.*, 1994) and focused radiotherapy (Mehta *et al.*, 1994) are possibly associated with improved survival but cure is likely to remain elusive with these techniques alone because of the adverse growth characteristics of gliomas (Kelly *et al.*, 1987). Chemotherapy offers the possibility of entire regional treatment but its impact on glioma therapy has been modest (Fine *et al.*, 1993). This is due to a number of factors including penetration problems, the intrinsic lack of efficacy of the available agents (Kornblith and Walker, 1988), the heterogeneity of tumours (Allalunis *et al.*, 1992) and adverse conditions in the associated microenvironment (Rampling *et al.*, 1994). There is a clear need for alternative therapies.

One approach has been to use viruses, either to deliver a therapeutic gene (Oldfield *et al.*, 1993) or as killing agents in their own right (Martuza *et al.*, 1991). This latter approach has particular appeal in the central nervous system (CNS) where the proliferative activity of the tumour distinguishes it from the surrounding normal brain which is essentially non-proliferative. Effective viral therapy in rodents based on replication-deficient mutants of herpes simplex virus type 1 (HSV-1) have been described (Mineta *et al.*, 1995).

Herpes simplex virus is a large double-stranded DNA virus whose genome consists of two unique segments, long and short, each bounded by a set of inverted repeats. The long repeat region of the genome contains a diploid gene, RL1 (Chou and Roizman, 1986; Dolan *et al.*, 1992) whose protein product ICP34.5 is a major determinant of pathogenicity (Chou *et al.*, 1990; MacLean *et al.*, 1991; McKie *et al.*, 1994). We have previously isolated an HSV-1 mutant 1716 which has a 759 bp depletion in both copies of RL1 (MacLean *et al.*, 1991). This mutant has a markedly attenuated ability to replicate in the CNS following both corneal and footpad inoculation of mice, and has an $LD_{50}$ $10^6$-fold higher than its parental strain $17^+$ following intracerebral inoculation. *In vitro* 1716 and other RL1 null mutants replicate as efficiently as their parental virus in established dividing cell lines from different species but replicate poorly in non-dividing cells (MacLean *et al.*, 1991; Brown *et al.*, 1994*a*).

It has previously been shown in one human neuroblastoma cell line SK-N-SH that mutants which fail to express ICP34.5 grow poorly and are specifically unable to prevent shut-off of cellular protein synthesis (Chou and Roizman, 1992), whereas infection of Vero cells which are non-neuronal in origin results in sustained protein synthesis and production of infectious progeny. Therefore in cells of neuronal origin ICP34.5 provides a survival mechanism which enables the virus to sustain its replication cycle and produce infectious virus. In the context of brain tumour therapy, RL1 deletion mutants have been identified as being of potential use since they should replicate preferentially in tumour cells, which constitute a mass of dividing cells within an otherwise quiescent population, non-permissive for viral replication. RL1 null mutants have the additional advantage in some cell lines that they provide a double hit phenomenon whereby cell killing should occur not only through lysis owing to growth and release of replicating virus, but also by premature shut-off of host cell protein synthesis. However, since the requirement of HSV-1 for ICP34.5 is both cell type- and

Correspondence: EA McKie
Received 1 December 1995; revised 12 March 1996; accepted 14 March 1996

cell state-specific (Brown et al., 1994a), it cannot be assumed at this stage that RL1 mutants will provide effective killing of all tumour cell types.

The therapeutic potential of RL1 mutants has so far been assessed in xenograph tumour models in animals (Markert et al., 1993; Chambers et al., 1995; Randazzo et al., 1995). Of necessity these model systems, although providing important information, cannot mimic the human situation where human heterogeneity and host environment can influence tumour and treatment behaviour. We therefore considered it important to determine whether RL1-negative HSV could kill naturally occurring human CNS tumours. To evaluate the potential efficacy of RL1 deletion mutants in the treatment of malignant glioma we have analysed viral replication *in vitro* in biopsy material obtained from patients with anaplastic astrocytoma or glioblastoma multiforme. In addition, we have assessed quantitatively the ability of the virus to grow in established human glioma cell lines *in vitro*. Owing to the inability to establish and grow sufficient numbers of cells, we were unable to obtain cultures of normal human brain tissue in sufficient quantities for comparative growth analysis.

### Materials and methods

### *Cells*

Baby hamster kidney clone 13 cells [BHK-21(C13); MacPherson and Stoker, 1962] were propagated in Eagle medium containing twice the normal concentration of vitamins and amino acids, 5% tryptose phosphate broth and 10% (v/v) fetal calf serum (FCS). The established human glioma cell lines used in this study are outlined in Table I. T98G, SB18, U-373MG, U-87MG, U251 were obtained from the European Tissue Culture Collection; MCN(X) was derived in the Medical Oncology Department and was obtained from Dr Pilkington, London. All established cell lines were propagated in a 50:50 mixture of Ham's F-10 medium and Dulbecco's modified Eagle medium (DMEM) containing 10% FCS, 5% L-glutamine and 50 $\mu$g ml$^{-1}$ gentamicin. To establish primary glioma cultures *in vitro*, glioma tissue was excised from patients undergoing cranial surgery in accordance with ethical procedures and a portion was placed immediately into ice-cold modified DMEM lacking serum; DMEM-BS (Bottenstein and Sato, 1979) containing 25 $\mu$g ml$^{-1}$ gentamicin. Tissue was not screened for presence of virus. The tumour tissue was finely chopped and collagenase (13.3 mg ml$^{-1}$; ICN Biochemicals, USA) mixed 1:1 (v:v) with L15 medium (Gibco) was added. The collagenase/tumour mixture was incubated for 30 min at room temperature and pipetted up and down with a 19-gauge needle to dissociate the tissue into single cells. The resulting cell suspension was washed in L15 medium and spun down at 2000 r.p.m. for 5 min. The cell pellet was split and resuspended in either 4 ml of DMEM-BS medium containing 1:1 (v:v) medium conditioned for type 1 astrocytes (Noble and Murray, 1984) or DMEM-BS containing 20% FCS; both cultures containing 25 $\mu$g ml$^{-1}$ gentamicin. Cells in the appropriate media were incubated on 25 cm$^3$ tissue culture flasks coated with 13 $\mu$g ml$^{-1}$ poly-L-lysine (Sigma) and incubated in 7% carbon dioxide at 37°C. For subsequent analysis of viral growth characteristics, cultures were expanded in a 50:50 mixture of DMEM and Ham's F-10

**Table I** Origin of cell lines tested for HSVI wild-type and RL1 mutant 1716 replication

| *Cell line* | *Origin* |
|---|---|
| Established cell lines | |
| MCN(X) | Human adult glioma passaged *in vivo* in mice; cell line established *in vitro* |
| T98G, SB18 U251 | Human glioblastoma multiforme |
| U-87MG, U-373MG | Human glioblastoma astrocytoma |
| Cell lines derived from human tumour biopsy material | |
| BG557, BG398, BG448 G-AST | Anaplastic astrocytoma |
| BG535, BG560, BG500 BG550, BG555 | Glioblastoma multiforme |

**Figure 1** Growth kinetics of HSV-1 strain 17$^+$ (-■-) and the RL1-negative mutant 1716 (....▲....) SB18, T98G and U251 cells were infected at a multiplicity of infection (m.o.i.) of 10 p.f.u. per cell (upper graph) or 0.001 p.f.u. per cell (lower graph), and at various times after infection the infected cells were harvested. The virus was released by sonication and titrated on BHK-21 (C13) cells at 37°C: (**a**) SB18 cells; (**b**) T98G cells and (**c**) U251 cells. Graphs shown are accurate representations of results obtained from more than one assay.

medium with antibiotics as above. Table I gives a summary of the origins of the cell lines established.

### Viruses

Virus stocks were grown and titrated on BHK21/C13 cells as previously described (Brown *et al.*, 1973). The parental HSV-1 strain used in this study was Glasgow strain $17^+$ (Brown *et al.*, 1973) and the RL1 mutant was 1716 (MacLean *et al.*, 1991)

### *Virus growth properties* in vitro

Approximately $2 \times 10^6$ cells were infected either at a multiplicity of 10 plaque-forming units (p.f.u.) per cell (single cycle) or 0.001 p.f.u. per cell (multiple cycles) with parental strain $17^+$ or RL1 mutant 1716. After absorption for 45 min at 37°C, the monolayers were washed, overlaid with the appropriate medium and incubated at 37°C. At intervals up to 24 h (single cycle) or 72 h (multiple cycles) after infection, samples were harvested and virus released by sonication was titrated on BHK21/C13 cells. Growth experiments were carried out in all the cell lines listed above.

### Immunofluorescence

Linbro wells containing coverslips were seeded with approximately $5 \times 10^4$ cells and incubated at 37°C overnight. Cells on coverslips were mock infected or infected at a multiplicity of infection (m.o.i.) of 10 p.f.u. per cell with parental or mutant viruses. Following incubation at 37°C for 10 h, cells were fixed with 4% paraformaldehyde for 15 min at room temperature, washed twice with phosphate-buffered saline (PBS) and stored in 70% ethanol at −20°C until use.

Coverslips were incubated initially with the monoclonal antibody Z1F11, which recognises the HSV-1 65K DNA-binding protein (kindly provided by Dr H Marsden); fluorosciene isothiocyanate conjugated goat anti-mouse IgG (Southern Biotechnique, Europath) was used as a second antibody. In some experiments cells were also stained with a rabbit polyclonal antiserum (Southern Biotechnique, Europath) against the glial fibrillary acidic protein (GFAP) (Southern Biotechnique, Europath) an astrocytic specific marker. In this case, rhodamine-conjugated goat anti-rabbit IgG (Europath) was used as a second antibody. Cells were viewed using a Nikon Microphot SA microscope.

### Labelling of infected cells with [$^{35}S$]methionine

Linbro wells containing either BG398, BG500 or BG560 cells were separately infected at a m.o.i. of 10 p.f.u. per cell with HSV-1 strain $17^+$ and 1716. At various times after infection, the cells were washed twice with PBS and the media replaced with PBS containing 50 $\mu$Ci $ml^{-1}$ [$^{35}$S]methionine. Labelling was continued for 1 h after which time the cells were harvested and protein extract from $2 \times 10^5$ cells separated using 7.5% SDS-PAGE as previously described (Marsden *et al.*, 1978).

### Results

### Growth characteristics of HSV-1 strain 17 and the mutant 1716 in established human glioma cell lines

In BHK21/C13 cells, which are routinely used for propagation and titration of HVS-1, wild-type virus and RL1 deletion mutants grow with indistinguishable kinetics at both high and low multiplicities of infection (MacLean *et al.*, 1991; McKie *et al.*, 1994). However, this phenotype is not reflected in all cell lines; in 3T6 mouse embryo fibroblasts, where wild-type HSV-1 replicates normally, RL1 mutant viruses are replication deficient (Brown *et al.*, 1994*a*, *b*); in F9 mouse teratocarcinoma stem cells, the phenotype is reversed and RL1 mutant virus replicates more efficiently than the wild-type virus (Brown *et al.*, 1994*a*). When F9 cells are induced to differentiate into parietal ectoderm by the addition of retinoic acid and dibutyryl cAMP, their permissivity for RL1 mutant virus increases while also becoming permissive for wild-type viral replication. It is evident therefore that both cell type and cell state determine the phenotype of HSV-1 variants which fail to synthesise ICP34.5. In order to evaluate efficiently the potential therapeutic value of RL1 mutant virus for the treatment of naturally occurring human brain tumours it became important to determine whether viral replication and cell killing was a predominant response in a wide range of tumour and established glioma cell lines *in vitro*.

Preliminary experiments were carried out at both high and low multiplicities of infection. Figure 1 shows the results of single and multi-cycle growth experiments with an initial infecting multiplicity of either 10 or 0.001 p.f.u. per cell. The cell lines used were the established human glioma cell lines T98G, SB18 and U251. In all three cases the growth patterns of wild-type and mutant virus were similar with a $10^2$- to $10^3$-fold increase in titre during the exponential phase of growth at high multiplicities of infection. However, in all cases growth of 1716 lagged slightly behind that of wild-type, subsequently leading to a lower final yield of virus. At lower multiplicities of infection, which determine the ability of the virus to be released

**Figure 2** Growth kinetics of HSV-1 strain $17^+$ (-■-) and the RL1-negative mutant 1716 (-▲-). (**a**) U-373MG and (**b**) U-87MG cells were infected at an m.o.i. of 10 p.f.u. per cell and at various times after infection the infected cells were harvested. The virus was released by sonication and titrated on BHK-21 (C13) cells at 37°C. Subsequent experiments demonstrated almost identical results.

from infected cells and undergo further rounds of replication, both mutant and wild-type viruses showed similar growth kinetics in all cell lines tested although the final titre of 1716 was reduced compared with the wild-type infection.

Several other established human glioma cell lines were tested for their ability to sustain viral replication at high multiplicities of infection (Figure 2). These included MCN(X), U-87MG and U-373MG. In both MCN(X) and U-373MG 1716 and $17^+$ grew efficiently, showing $10^2$- to $10^3$-fold increases in titre during the exponential phase of growth. In U-87MG cells, $17^+$ also showed a $10^2$- to $10^3$-fold increase in titre during the exponential phase of growth, whereas 1716 showed limited replication in this cell line. Interestingly, both viruses failed to replicate in MCX(X) cells (data not shown) and unlike primary tumour cells which were non-permissive for HSV (e.g. BG500) this cell line showed no signs of viral c.p.e.

### *Growth of wild-type and RL1 mutant virus in tumour biopsy material*

Having established that the RL1-negative mutant virus 1716 can replicate efficiently and destroy established human glioma cell lines *in vitro*, we wished to determine whether it was also effective in killing tumour cells obtained from patients undergoing cranial surgery. In all cases, cells were passaged as few times as possible to maintain their phenotype and limit selection for a subpopulation of rapidly growing cells. All cells were used at pass numbers between 2 and 4.

A total of nine tumour-derived cell lines from patients diagnosed as having either anaplastic astrocytoma or glioblastoma multiforme were tested for their ability to sustain viral replication. Owing to the limited numbers of cells available, virus growth experiments were only carried out at high multiplicities of infection (Figure 3). Several 24 h yield experiments were carried out for each cell line, where only the titre of input virus and yield at 24 h after infection were calculated; these demonstrated identical fold increases in titre to those presented in the graphs. In general, both wild-type and mutant virus replication was observed, with increases in titres ranging from 10-fold to 3000-fold during the exponential phase of growth. In one cell line, BG500, 1716 failed to replicate, whereas the titre of $17^+$ increased ~20-fold during the exponential phase of growth.

### *Viral antigen expression in permissive and non-permissive cultures*

To establish whether lack of 1716 replication in certain cell lines was blocked at a stage before or after viral entry, viral antigen expression was detected by immunofluorescence. We studied BG500 cells, as a representative tumour-derived cell line that was non-permissive for 1716, and MCN(X) the only established glioma cell line tested that was totally non-permissive for HSV-1. Cell lines that were either fully-permissive (BG398) or semipermissive (BG560) for 1716 replication were used as controls.

**Figure 3** Growth kinetics of HSV-1 strain $17^+$ (-■-) and the RL1-negative mutant 1716 (-▲-). BG500 (**a**), G-AST (**b**), BG398 (**c**), BG448 (**d**), BG550 (**e**), BG560 (**f**), BG535 (**g**), BG557 (**h**) and BG555 (**i**) cells were infected at an m.o.i. of 10 p.f.u. per cell and at various times after infection the infected cells were harvested. The virus was released by sonication and titrated on BHK-21(C13) cells at 37°C. In cases where sufficient cells could be obtained to examine viral growth kinetics more than once final viral titres could be accurately reproduced.

Cells were infected as described in Materials and methods, fixed 10 h after infection and stained with Z1F11, a monoclonal antibody directed against the HSV-1 early protein 65K (Figure 4b and 4d). The percentage of cells expressing 65K was determined for each cell line. As predicted from the growth experiments, 1716-infected BG398 cultures displayed a higher proportion of antigen-positive cells (79%) than mutant-infected BG560 (48%) or BG500 (34%). In all cultures infected with wild-type virus, 85–95% of cells were infected at this time point and viral yield appeared to be directly related to infectivity. There were no antigen-positive MCN(X) cells in cultures infected with either wild-type virus or 1716, demonstrating that HSV is unable to initiate replication in these cells. Interestingly, MCN(X) cells were also negative for GFAP by immunofluorescence staining.

To determine whether there was a correlation between expression of GFAP and permissivity for HSV-1, the immunofluoresence experiments were repeated and cells were labelled for expression of both GFAP (Figure 4a and c) and 65K. We were unable to demonstrate a correlation between GFAP expression and HSV permissivity, as BG398 cells, which were highly permissive for both wild-type and 1716 replication had a low percentage of GFAP positive cells (14%) whereas BG500 which were non-permissive for 1716, but permissive for $17^+$ had a high percentage of GFAP-expressing cells (75%). There was no correlation between expression of GFAP in any individual cell and permissivity for HSV, as all cultures contained 65K-positive cells which were either GFAP-positive or GFAP-negative.

*Failure of RL1 mutants to replicate in some cell lines is caused by premature shut-off of host cell protein synthesis*

Having established in BG500 cells that the block in the viral replication cycle is not caused by an inability of the virus to enter the cell, we wished to determine whether the failure to support replication of RL1 mutant virus was due to shut-off of cellular protein synthesis. Three cell lines were selected which varied in their permissivity for 1716: BG398, which was fully permissive; BG560, which was semipermissive; and BG500, which was non-permissive. Figure 5 shows the levels of [$^{35}$S]methionine incorporated into cells infected with either wild-type or 1716 or uninfected controls.

In fully permissive BG398 cells the levels of cellular protein synthesis are very similar following infection with either wild-type or 1716 at all time points examined although there appears to be a slight decrease in protein synthesis by 8–9 h after infection in cells infected with 1716. In cell lines which are less permissive for 1716 compared with $17^+$ there are reduced levels of protein synthesis in 1716-infected cells harvested as early as 5–7 h after infection; 9 h after infection, incorporation of [$^{35}$S]methionine is markedly reduced in BG500 and BG560 cells.

### Discussion

Several studies in mice have demonstrated regression of experimentally implanted tumours by replication-compromised variants of herpes simplex virus that replicate normally in dividing cells but fail to replicate in non-dividing cells of the nervous system. One of the earliest studies using a thymidine kinase (TK)-negative HSV mutant dlsptk (Coen et al., 1989) demonstrated a dose-dependent improvement in survival of nude mice bearing intracranial tumours following intratumoral therapy (Martuza et al., 1991). However, subsequent studies demonstrated that this mutant is unsuitable for therapeutic use in humans as it can cause encephalitis.

RL1 variants have been shown to improve the survival of nude mice bearing intracranial human gliomas (Markert et al., 1993; Chambers et al., 1995; Kesari et al., 1995) and have the added advantage that they are totally non-neurovirulent (Chou and Roizman, 1990; MacLean et al., 1991; McKie et al., 1994). A recent study using the Harding–Passey melanoma cell line to establish CNS tumours in C57Bl/6 mice demonstrated that stereotactic injection of the avirulent HSV variant 1716 into the tumour 5–10 days after CNS

**Figure 4** Localisation of the HSV-1 65 kDa DNA-binding protein and GFAP in infected cells. These photographs are representative examples of (**a**) $17^+$-infected BG398 cells incubated with anti-GFAP; (**b**) 1716-infected BG398 cells incubated with Z1F11; and 1716-infected BG500 cells incubated with either (**c**) anti-GFAP or (**d**) Z1F11.

**Figure 5** Autoradiographs of electrophorectically separated lysates of infected cells labelled for 60 min with [$^{35}$S]methionine. (**a**) BG398, (**b**) BG560 and (**c**) BG500 cells in linbro wells were either mock-infected or infected at an m.o.i. of 10 p.f.u. per cell with $17^+$ or the RL1-negative mutant 1716. At various times after infection (p.i.) the cells were harvested, lysed and extracts separated by 7.5% SDS-PAGE. Extracts from $2 \times 10^5$ cells are loaded in each lane. For each cell line; 1, mock, 1 h p.i.; 2, $17^+$, 3 h p.i.; 3, 1716, 3 h p.i.; 4, $17^+$, 5 h p.i.; 5, 1716, 5 h p.i.; 6, $17^+$, 7 h p.i.; 7, 1716, 7 h p.i.; 8, $17^+$, 8 h p.i.; 9, 1716, 8 h p.i.; 10, $17^+$, 9 h p.i.; 11, 1716, 9 h p.i.; 12, mock, 9 h p.i.

seeding of the melanoma cells results in a significant increase in the time to development of neurological symptoms, complete tumour regression and long-term survival of animals (Randazzo *et al.*, 1995). One of the most relevant findings from this study in terms of safety of RL1 mutants was the demonstration that in mice treated with 1716, HSV antigen staining was contained in the tumour mass with no spread to adjacent tissue or distant regions of the brain. These results substantiated previous studies which showed that following intracerebral inoculation 1716 fails to replicate and hence fails to kill either BALB/c (MacLean *et al.*, 1991) or immunocompromised SCID mice (Valyi-Nagy *et al.*, 1994); a recent study has also demonstrated that an HSV-1 variant containing a mutation in RL1 is similarly non-neurovirulent following intracranial inoculation of owl monkeys (Mineta *et al.*, 1995) and we would also predict that RL1 mutants will fail to replicate in the human CNS.

Previous studies which have used herpes simplex virus vectors for the treatment of experimentally induced brain tumours have been directed towards the development of relevant *in vivo* models to study tumour regression and long-term survival. Consequently, these studies have concentrated on cell lines which are known to grow as xenographs in mice (Markert *et al.*, 1993; Chambers *et al.*, 1995) with very little quantitative analysis of viral replication in established CNS tumour cell lines or primary tumour tissue obtained from surgical specimens. If RL1-negative HSV is to be seriously considered as a realistic tumour therapy, it is crucial to ensure that the virus is capable of replicating and killing naturally occurring tumours. Therefore, as a preliminary prerequisite to human therapy, we have evaluated the CNS tumour killing potential of RL1-negative HSV.

With the exception of one cell line, MCN(X), we found that wild-type HSV-1 replicated in all cell lines tested. In general wild-type and mutant virus showed similar replication kinetics in cell lines derived from anaplastic astrocytoma (e.g. G-AST and BG398), but were more variable in cells derived from glioblastoma multiforme (e.g. BG500 and BG560). This could be explained by the heterogeneous pathology of glioblastoma multiforme, which could include populations of refractile cells non-permissive for HSV. Immunofluorescence staining of MCN(X) demonstrated that viral gene expression was not initiated by either wild-type virus or 1716 in these cells and unlike BG500 cells, which were also non-permissive for 1716, these cells failed to round up and die. The survival of MCN(X) cells following infection supports the double-hit response hypothesis as these cells do not suffer an infection-induced death. The failure of HSV to replicate in this cell line could be owing to the lack of suitable cellular receptors for adsorption and/or penetration or it could be at a stage subsequent to virus entry.

1716 also failed to replicate in one cell line derived from tumour biopsy material. In this case the mutant entered the cell as indicated by expression of the 65K DNA-binding protein yet no infectious virus was produced possibly as a consequence of the shut-off of cellular protein synthesis demonstrated early in infection. However, a limited number of cells did support 1716 replication, a situation similar to that recently demonstrated in 3T6 cells (Brown *et al.*, 1994*a*). One possible explanation for this finding is that a proportion of the cells produce a protein which can compensate for the loss of ICP34.5, preventing shut-off of cellular protein synthesis thus allowing viral replication. However the decline in protein synthesis observed in both BG500 and BG560 cells infected with 1716 appeared similar, yet BG500 cells were totally non-permissive for 1716 and a direct correlation cannot be made between viral titre and degree of host cell protein synthesis shut-off–other factors are certainly involved. Variations in expression of proteins which are functionally homologous to ICP34.5 could account for the differences observed in 1716 growth or alternatively the cellular proteins could be totally unrelated. As the cells were infected at high multiplicities of infection, where all cells in the culture would be expected to be infected, a difference in individual cellular metabolism or protein expression would seem a more likely explanation than a defect in maturation and virus egress preventing further rounds of replication as previously shown in 3T6 (Brown 1994*b*).

The finding that not all tumour cells are permissive for 1716 replication is important not only in terms of the biology of HSV but also in its implications for the treatment of human gliomas. At face value it indicates that not all human gliomas could be successfully treated using HSV RL1 null mutant therapy. The BG500 cell line was derived from a patient with glioblastoma multiforme, the most commonly found primary tumour of the nervous system. As indicated by the term 'multiforme', the tumour is characterised by a pleomorphic cellular population. Chromosome banding techniques (Bigner *et al.*, 1984, 1986) have indicated that the tumour may evolve from a single malignant progenitor cell and that the phenotypic and genotypic heterogeneity observed is a result of secondary changes. Therefore, in reality, every patient who presents with this form of glioma may have a completely different tumour cell composition which consequently may vary greatly in permissivity for RL1 null mutant replication.

We have shown that 1716 replicates ~100-fold less efficiently than wild-type virus in U87-MG cells *in vitro*. In a recent study Mineta *et al.* (1995) used the U-87MG cell line to implant tumours intracerebrally into mice and demonstrated complete tumour regression in this model when animals were injected with $10^7$ p.f.u. of G207, a multi-mutated variant of HSV-1 strain F which fails to synthesise both ICP34.5 and ribonucleotide reductase. These complementary *in vivo* and *in vitro* findings suggest that even limited viral replication could be sufficient to enable complete tumour regression in humans.

As demonstrated, infection with RL1-negative virus which does not result in lytic replication in specific cell types can trigger the host cell into premature protein synthesis shut-off. Hence, although the tumour cells are not killed by virus replication, the cells could die owing to cessation of protein synthesis. HSV, RL1 mutant therapy has therefore the distinct advantage of a double-hit response. The added potential of introducing a transgene capable of expressing a tumour-killing agent into RL1-negative HSV is obvious. As the mode of infection in tumour cells is lytic as opposed to latent, long-term expression from a therapeutic gene would not be required.

**Acknowledgements**
EA McKie is employed on funds from Bayer UK to PGEK. Support for the research was provided by the MRC, the Wellcome Trust and the Cancer Research Campaign.

### References

ALBERT FK, FORSTING F, SARTOR K, ADAMS HP AND KUNZE S. (1994). Early post-operative magnetic resonance imaging after resection of malignant glioma: objective evaluation of residual tumour and its influence on regrowth and prognosis. *Neurosurgery*, **34**, 445–461.

ALLALUNIS TMJ, BARRON GM, DAY RS III, DOBLER K AND URTASUN RC. (1992). Heterogeneity in response to treatment with butathione sulfoximine or interferon in human malignant glioma cells. *Int. J. Radiat. Oncol. Biol. Phys.*, **22**, 765–768.

BIGNER SH, MARK J, MAHALEY MS AND BIGNER DD. (1984). Patterns of the early gross chromosomal changes in malignant human gliomas. *Hereditas*, **101**, 103–113.

BIGNER SH, MARK J, BULLARD DE, MAHALEY MS Jr AND BIGNER DD. (1986). Chromosomal evolution in malignant human gliomas starts with specific and usually numerical deviations. *Cancer Genet. Cytogenet.*, **22**, 121–135.

BOTTENSTEIN JE AND SATO GH. (1979). Growth of a rat neuroblastoma cell line in serum-free supplemented medium. *Proc. Natl Acad. Sci. USA*, **76**, 514–517.

BROWN SM, RITCHIE DA AND SUBAK-SHARPE JH. (1973). Genetic studies with herpes simplex virus type 1. The isolation of temperature sensitive mutants, their arrangement into complementation groups and recombination analysis leading to a linkage map. *J. Gen. Virol.*, **18**, 329–346.

BROWN SM, HARLAND J, MACLEAN AR, PODLECH J AND CLEMENTS JB (1994*a*) Cell type and cell state determine differential *in vitro* growth of non-neurovirulent ICP34.5 negative herpes simplex virus. *J. Gen. Virol.*, **75**, 2367–2377.

BROWN SM, MACLEAN AR, AITKEN JD AND HARLAND J. (1994*b*). IP34.5 influences herpes simplex virus type 1 maturation and egress from infected cells *in vitro*. *J. Gen. Virol.*, **75**, 3679–3686.

CHAMBERS R, GILLESPIE GY, SOROCEANU L, ANDREANSKY S, CHATTERJEE S, CHOU J, ROIZMAN B AND WHITLEY RJ. (1995). Comparison of genetically engineered herpes simplex viruses for the treatment of brain tumors in a scid mouse model of human malignant glioma. *Proc. Natl Acad. Sci. USA*, **92**, 1411–1415.

CHANG CH, HORTON J, SCHOENFELD D, SALAZAR O, PEREZ-TAMAYO R, KRAMER S, WEINSTEIN A, NELSON JS AND TSUKADA Y. (1983). Comparison of post-operative radiotherapy and combined post-operative radiotherapy and chemotherapy in the multidisciplinary management of malignant gliomas. *Cancer*, **52**, 997–1007.

CHOU J AND ROIZMAN B. (1986). The terminal a sequence of the herpes simplex virus genome contains the promoter of a gene located in the repeat sequences of the L component. *J. Virol.*, **57**, 629–637.

CHOU J AND ROIZMAN B. (1992). The $\gamma$34.5 gene of herpes simplex virus type 1 precludes neuroblastoma cells from triggering total shutoff of protein synthesis characteristic of programmed cell death in neuronal cells. *Proc. Natl Acad. Sci. USA*, **89**, 3266–3270.

CHOU J, KERN ER, WHITLEY RJ AND ROIZMAN B. (1990). Mapping of herpes simplex virus type 1 neurovirulence to ICP34.5, a gene non-essential for growth in culture. *Science*, **250**, 1262–1265.

COEN DM, KOSZ-VNENCHAK M, JACOBSON JG, LEIB DA *et al.* (1989). Thymidine kinase-negative herpes simplex virus mutants establish latency in mouse trigeminal ganglia but do not reactivate. *Proc. Natl Acad. Sci. USA*, **86**, 4736–4740.

DOLAN A, MCKIE EM MACLEAN AR AND MCGEOCH DJ. (1992). Status of the ICP34.5 gene in herpes simplex virus type 1 strain 17. *J. Gen. Virol.*, **73**, 971–973.

FINE H, DEAR KB, LOEFFLER JS, BLACK P AND CANELLOS GP. (1993). Meta-analysis of radiation therapy with or without adjuvant chemotherapy for malignant gliomas in adults. *Cancer*, **71**, 2585–2597.

KELLY PJ, DUMAS-DUPORT C, KISPERT DB, KALL BA, SCHEITHAUER BW AND ILLIG JJ. (1987). Imaging based stereotaxic serial biopsies in untreated intracranial glial neoplasms. *J. Neurol.*, **66**, 865–874.

KESARI S, RANDAZZO BP, VALYI-NAGY T, HUANG QS, BROWN SM, MACLEAN AR, LEE VM-Y, TROJANOWSKI JQ AND MACKIE T. (1995). A mutant herpes simplex virus replicates in brain tumours but not in neurons derived from a human embryonal carcinoma cell line. *Lab. Invest.*, **73**, 636–648.

KORNBLITH PL AND WALKER M. (1988). Chemotherapy for malignant gliomas. *J. Neurol.*, **68**, 1–17.

MCKIE EA, HOPE RG, BROWN SM AND MACLEAN AR. (1994). Characterization of the herpes simplex virus type 1 strain $17^+$ neurovirulence gene RL1 and its expression in a bacterial system. *J. Gen. Virol.*, **75**, 733–741.

MACLEAN AR, FAREED MU, ROBERTSON L, HARLAND J AND BROWN SM. (1991). Herpes simplex virus type 1 deletion variants 1714 and 1716 pinpoint neurovirulence related sequences in Glasgow strain $17^+$ between immediate early gene 1 and the 'a' sequence. *J. Gen. Virol.*, **72**, 631–639.

MACPHERSON I AND STOKER MG. (1962). Polyoma transformation of hamster cell clones: an investigation of genetic factors affecting cell competence. *Virology*, **16**, 147–151.

MARTUZA RL, MALICK A, MARKERT JM, RUFFNER KL AND COEN DM. (1991). Experimental therapy of human glioma by means of a genetically engineered virus mutant. *Science*, **252**, 854–856.

MARKERT JM, MALICK A, COEN DM AND MARTUZA RL. (1993). Reduction and elimination of encephalitis in an experimental glioma therapy model with attenuated herpes simplex mutants that retain susceptibility to acyclovir. *Neurosurgery*, **32**, 597–603.

MARSDEN HS, STOW ND, PRESTON VG, TIMBURY MC AND WILKIE NM. (1978). Physical mapping of herpes simplex virus-induced polypeptides. *J. Virol.*, **28**, 624–642.

MEHTA MP, MASCIOPINTO J, ROSENTHAL J, LEVIN A, CHAPPELL R, BASTIN K, MILES J, TURSKI P, KUBSAD S AND MACKIE T. (1994). Stereotactic radiosurgery for glioblastoma multiforme; report of a prospective study evaluating prognostic factors and analysing long term survival advantage. *Int. J. Oncol. Biol. Phys.*, **30**, 541–549.

MINETA T, RABKIN SD AND MARTUZA RL. (1994). Treatment of malignant gliomas using ganciclovir-hypersensitive, ribonucleotide reductase-deficient herpes simplex viral mutant. *Cancer Res.*, **54**, 3963–3966.

MINETA T, RABKIN SD, YAZAKI T, HUNTER WD AND MARTUZA RL. (1995). Attenuated multi-mutated herpes simplex virus-1 for the treatment of malignant gliomas. *Nature Med.*, **1**, 938–943.

NOBLE M AND MURRAY K. (1984). Purified astrocytes promote the division of a bipotential glial progenitor cell. *EMBO J.*, **3**, 2243–2247.

OLDFIELD EH, RAM Z, CULVER K, BLAESE RM, DE VROOM HL AND ANDERSON WF. (1993). Gene therapy for treatment of brain tumors using intra-tumoral transduction with thymidine kinase gene and intravenous ganciclovir. *Human Gene Therapy*, **4**, 39–69.

RAMPLING R, CRUICKSHANK G, LEWIS AD, FITZSIMMONS SA AND WORKMAN P. (1994). Direct measurement of $pO_2$ distribution and bioreductive enzymes in human malignant brain tumours. *Int. J. Radiat. Oncol. Biol. Phys.*, **29**, 427–431.

RANDAZZO RBP, KESARI S, GESSER RM, ALSOP D, FORD JC, BROWN SM, MACLEAN AR AND FRASER NW. (1995). Treatment of experimental intracranial murine melanoma with neuroattenuated herpes simplex virus type 1 mutant. *Virology*, **211**, 94–101.

VALYI-NAGY T, FAREED MU, O'KEEFE JS, GESSER RM, MACLEAN AR, BROWN SM, SPIVAK JG AND FRASER NW. (1994). The HSV-1 strain $17^+$ gamma 34.5 deletion mutant 1716 is avirulent in SCID mice. *J. Gen. Virol.* **75**, 2059–2063.